# Physiological Emergency Surgery Acuity Score Predicts Mortality and Anastomotic Leak in Emergency Laparotomy: An External Validation Study

**DOI:** 10.1002/wjs.70562

**Published:** 2026-09-05

**Authors:** Wade Hopper, Anthony J. Duncan, Mentor Ahmeti

**Affiliations:** ^1^ School of Medicine & Health Sciences Department of Surgery University of North Dakota Grand Forks North Dakota USA; ^2^ Department of Surgery Division of Trauma and Surgical Critical Care Sanford Medical Center Fargo Fargo North Dakota USA

**Keywords:** anastomotic leak, general surgery, hospital mortality, laparotomy, postoperative complications, risk assessment

## Abstract

**Background:**

The Physiological Emergency Surgery Acuity Score (PESAS) predicts 30‐day mortality but has not been externally validated in emergency general surgery (EGS) patients. The study aim was to evaluate PESAS discrimination for predicting mortality and anastomotic leak in patients receiving emergency laparotomy.

**Methods:**

We retrospectively evaluated PESAS in a single‐center cohort of adults receiving exploratory laparotomy for emergent, nontraumatic indications. PESAS and 30‐day mortality were calculated for all patients. Discrimination of PESAS for 30‐day mortality was assessed using area under receiver operator curve (AUROC). Secondary analysis was conducted in similar fashion for the outcome of anastomotic leak requiring reoperation.

**Results:**

In 1086 patients with 160 cases of 30‐day mortality (14.7%), PESAS showed strong discrimination for the primary outcome (AUROC 0.86; 95% CI 0.83–0.88) with acceptable calibration‐in‐the‐large. In an exploratory secondary analysis of 414 patients who received bowel anastomosis with 21 cases of leak (5.1%), PESAS also showed good discrimination for anastomotic leak (AUROC 0.82; 95% CI 0.73–0.90). Observed mortality rates showed clear inflection points at PESAS values of 5 and 10, respectively, corresponding to mortality exceeding 10% and 50%.

**Conclusions:**

PESAS demonstrates good discrimination, calibration‐in‐the‐large, and net benefit as an ordinal risk stratification tool for prediction of 30‐day mortality among patients undergoing emergency laparotomy, with exploratory analysis suggesting promise for predicting anastomotic leak. Because it is derived entirely from objective physiologic variables, it may be well‐suited for automated risk stratification in EGS populations. Further patient‐level calibration is needed to justify expanded use.

## Graphical abstract

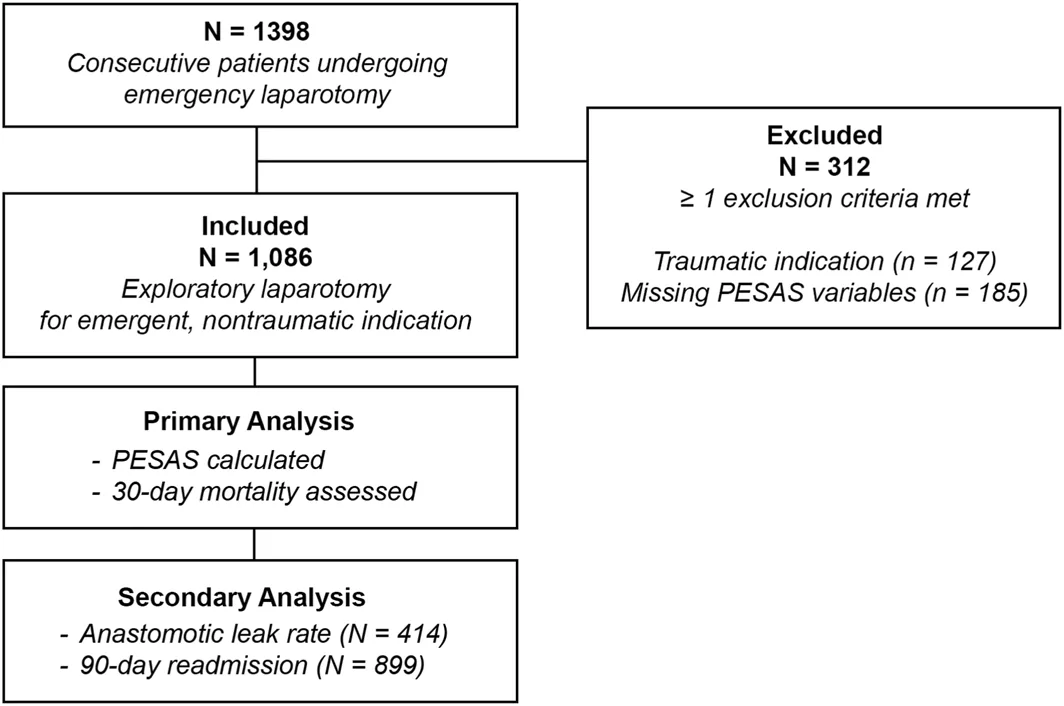

## 1 Introduction

Provider estimation of patient acuity is a key determinant of patient outcomes and resource expenditure [1]. Generally, acuity refers to risk for poor outcomes including mortality. Underestimation of acuity can delay care and lead to harm, while overestimation of acuity contributes to resource scarcity [1]. Emergency general surgery (EGS) is associated with high patient acuity, 30‐day mortality around 12%, and a major complication rate around 30% [2]. Several risk prediction models are currently used in the EGS context; however, no model performs optimally across all patient populations and outcomes [3]. Commonly used models include the American College of Surgeons National Quality Improvement Program (ACS‐NSQIP) calculator, Predictive Optimal Trees in Emergency Surgery Risk (POTTER), and Emergency Surgery Acuity Score (ESAS). Many models rely upon subjective input variables or require significant user interaction.

The Physiological Emergency Surgery Acuity Score (PESAS) predicts the risk of 30‐day postoperative mortality in patients undergoing emergency surgery [4]. It was developed in 2017 using a general population from the ACS‐NSQIP database involving a wide range of surgical subspecialties and patient demographics. PESAS input data is derived from routine blood chemistry studies in addition to whether or not patients are mechanically ventilated. It does not incorporate demographic data or comorbidities. Internal validation of PESAS was reported alongside its original derivation, but the score has not been externally validated within specific surgical subpopulations [4]. Use of the PESAS in EGS patients is accepted despite lack of robust evidence supporting its use in this demographic [3]. Therefore, the purpose of this study was to evaluate the external performance of PESAS for postoperative risk stratification of 30‐day mortality and anastomotic leak in patients undergoing emergency laparotomy.

## 2 Methods

### 2.1 Study Design and Population

This study received Institutional Review Board approval (STUDY00004011), and waiver of patient consent was obtained. The Transparent Reporting of a Multivariable Prediction Model for Individual Prognosis or Diagnosis (TRIPOD) checklist was completed to facilitate reporting (Supporting Information S1). We retrospectively applied the PESAS to a cohort of adult patients (age ≥ 18 years) who received exploratory laparotomy for emergent, nontraumatic indications at our quaternary care center between January 1, 2014 and January 17, 2024. Data for all consecutive patients receiving exploratory laparotomy within the study timeframe was extracted from retrospective review of electronic medical records (EMR) and compiled within an institutional laparotomy registry. Exploratory laparotomy was identified using CPT code 49000. Emergency cases were defined as those performed within 12 h of hospital admission or documented symptom onset; patients whose operation occurred outside this 12‐h window were not included in our institutional laparotomy registry. Exclusion criteria were laparotomy for traumatic indications, age less than 18 years, pregnant patients, and incarcerated patients. Additionally, patients missing ≥ 1 PESAS variable were excluded from analysis, and imputation of the composite score was not attempted. Deidentified data was compiled in REDCap [5].

### 2.2 Outcomes

The primary study outcomes were PESAS discrimination, calibration, and net benefit for predicting 30‐day mortality. The secondary outcome was PESAS discrimination for predicting anastomotic leak requiring reoperation among patients receiving bowel anastomosis. Discrimination was operationalized as area under receiver operator curve (AUROC). Calibration was operationalized as observed‐to‐expected (O/E) event ratio and visualized at the level of the individual PESAS score using a calibration curve with slope and intercept. Net benefit was calculated as the difference between true positive and false negative classifications, weighted by odds of threshold probabilities.

### 2.3 Predictors

Preoperative variables included in the PESAS were identified and the score was calculated for each patient as originally published [4]. Score variables included whether or not mechanical ventilation was required within 48 h preoperatively as well as serum levels of albumin, alkaline phosphatase, blood urea nitrogen, creatinine, international normalized ratio (INR), platelets, aspartate aminotransferase, sodium, and white blood cell count. Each laboratory component was taken from the most recent preoperative value, and each was characterized at the thresholds reported in the original manuscript [4]. The incidence of mortality within 30 days of index surgery was determined for each patient. American Society of Anesthesiologists physical status classification (ASA) and Charlson comorbidity index (CCI) scores were also calculated. The main operative diagnosis for each patient's index case was recorded. For each patient receiving a primary bowel anastomosis, the incidence of anastomotic leak requiring reoperation was calculated.

### 2.4 Sample Size

This study used all eligible patients available within our institutional laparotomy registry during the defined study timeframe, rather than a pre‐specified enrollment target. Sample size adequacy was assessed post hoc using the methods proposed by Riley et al. for outcomes of C‐statistic, O/E ratio, and net benefit [6]. For discrimination as measured by C‐statistic, assuming our cohort's mortality rate (14.7%) and an anticipated C‐statistic of 0.75 (0.05 below that of the derivation cohort), we estimated that 724 patients were required for adequate precision. For calibration‐in‐the‐large as measured by O/E, using our cohort's mortality rate and an expected O/E of 1 with a target 95% confidence interval (CI) width of 0.4, we estimated that 566 patients were required for adequate calibration‐in‐the‐large. For decision curve analysis, assuming our cohort's mortality rate and targeting a 95% CI width of 0.2 for standardized net benefit, the primary analysis was adequately powered at the 10% threshold (requiring 410 patients) and 15% risk threshold (requiring 694 patients), but not for a 25% risk threshold (requiring 1903 patients). Our cohort was underpowered for the secondary outcome of anastomotic leak, and secondary findings should be interpreted as exploratory.

### 2.5 Statistical Analysis

Categorical data was presented as numbers and percentages. Continuous data was presented as means and standard deviations or medians and interquartile ranges based on normality of distribution, assessed visually using histograms. Mortality data from the current cohort was compared against that reported by the published PESAS development cohort [4]. Two‐tailed 95% confidence intervals were calculated using the Wilson method. Univariable logistic regression analysis was performed with the outcome of 30‐day mortality and the independent variable of PESAS score. PESAS was analyzed as an ordinal integer score entered as a single term in logistic regression. Discrimination was assessed with the concordance statistic (*c*) equal to the AUROC of the model. The optimal predictive threshold was determined by maximal Youden's index. Calibration‐in‐the‐large was evaluated using the O/E mortality ratio with 95% Wilson confidence intervals. Calibration was visualized by plotting validation cohort mortality (observed risk) against derivation cohort mortality (predicted risk) for each PESAS score and using a sample‐size‐weighted least‐squares linear regression to obtain slope and intercept. Aggregate (score‐level) calibration was performed due to lack of access to the score derivation regression equation; therefore, results represent the transportability of derivation cohort risk estimates into a novel clinical setting, rather than calibration at the individual patient level. Clinical utility was assessed with decision curve analysis across threshold probabilities from 0% to 100% according to the method described by Vickers and Elkin [7]. For the secondary outcome of anastomotic leak, regression analysis was repeated but calibration and decision curve analyses were not performed. Data were visualized by clustering patients into 3 strata based on PESAS (< 5, 6–9, and ≥ 10) and calculating Wilson–Brown 95% confidence intervals for outcomes of mortality and anastomotic leak within each stratum. Within the validation cohort, discrimination of PESAS was compared against that of the ASA classification and the Charlson Comorbidity Index using DeLong's test. Statistical analysis and data visualization was performed in GraphPad Prism version 11.0.0 and R version 4.4.1 using the *dcurves* and *ggplot2* packages.

## 3 Results

A total of 1398 records were available for patients who underwent exploratory laparotomy at our institution within the study time frame. A study design flowchart is shown in Figure 1. Of the 312 excluded records, 127 were excluded for a traumatic operative indication and 185 were excluded for missing one or more PESAS component variables. There were 1086 patients who met inclusion criteria and were included in final analysis (Table 1), including 160 cases of 30‐day mortality (14.7%). The mean age of the population was 65.4 ± 16.4 years. The median PESAS score was 3 (interquartile range 1–6). The lowest observed PESAS score was 1, and no patients had a score of 0. The most common indications for surgery were small bowel obstruction (272 cases, 25%) and perforation (248 cases, 23%).

**FIGURE 1:**
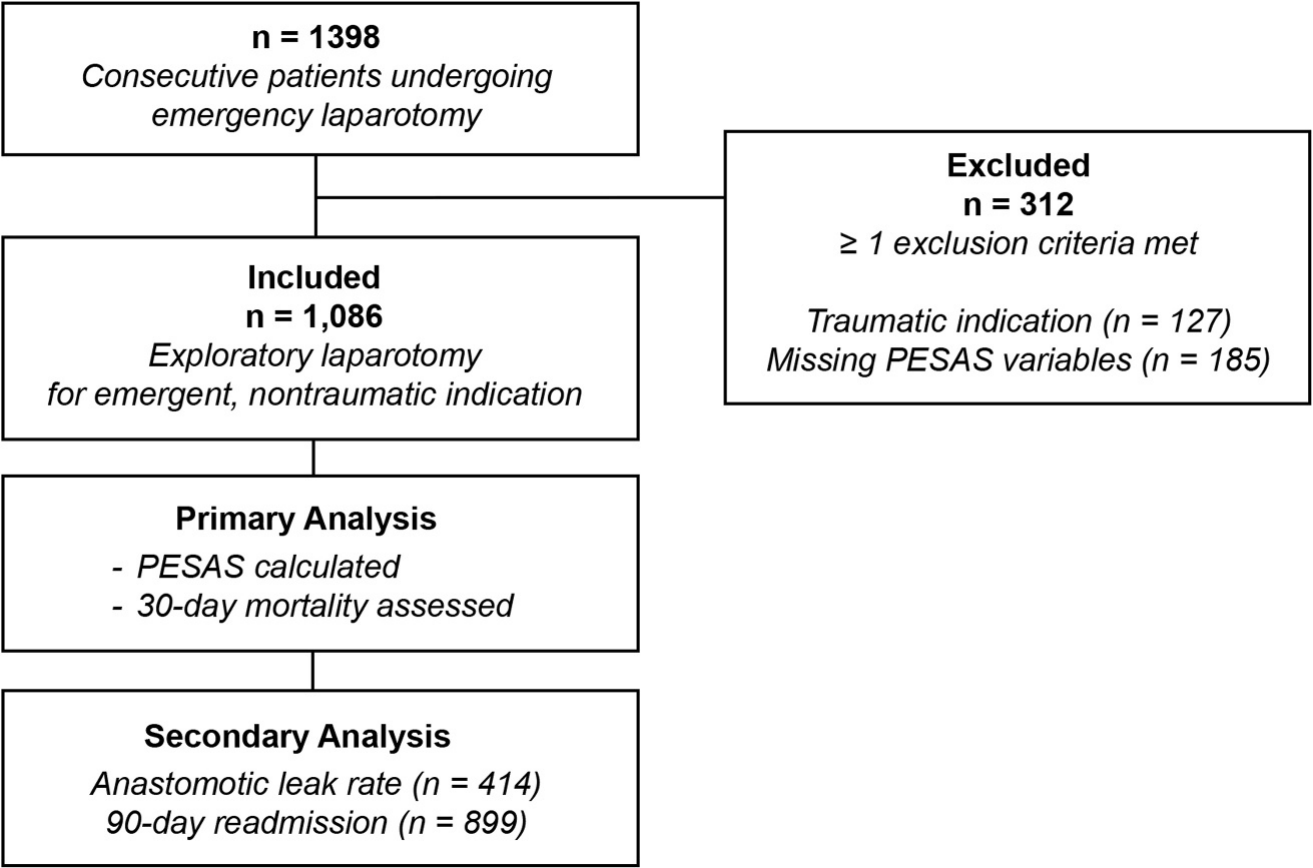
Study flowchart. PESAS, Physiological Emergency Surgery Acuity Score.

**TABLE 1:** Patient characteristics.

Variable	Validation cohort^a^
*N*	1086
Mortality	160 (14.7%)
Demographics	
Age > 65 years	598 (55.1%)
White race	992 (91.3%)
Female	562 (51.8%)
Transfer from outside facility	443 (40.8%)
Transfer from critical access hospital	225 (20.7%)
ASA	3 [3–4]
ASA 3	545 (50%)
ASA 4	272 (25%)
ASA 5	59 (5.4%)
CCI	3 [2–4]
PESAS	3 [1–6]
Level of care	
Floor	548 (50.5%)
Intermediate	84 (7.7%)
ICU	454 (41.8%)
Operative diagnosis	
Small bowel obstruction	272 (25.1%)
Large bowel obstruction	54 (5.0%)
Volvulus	89 (8.2%)
Perforation	248 (22.8%)
Mesenteric ischemia	168 (15.5%)
Incarcerated hernia	66 (6.1%)
Diverticulitis	132 (12.2%)
Other	57 (5.3%)

Abbreviations: ASA, American Society of Anesthesiology physical status classification; CCI, Charlson comorbidity index; ICU, Intensive care unit; PESAS, Physiological Emergency Surgery Acuity Score.

^a^ *n* (%) or median [interquartile range].

PESAS showed strong discrimination for 30‐day mortality with an AUROC of 0.86 (*p* < 0.001; 95% CI 0.83–0.88). The odds ratio per one‐point increase in PESAS was 1.61 (95% CI 1.50–1.73). The optimal threshold for prediction of 30‐day mortality was PESAS ≥ 5 with a sensitivity of 90.6% (95% CI 85.1%–94.2%) and a specificity of 66.3% (95% CI 63.2%–69.3%). At the optimal cutoff of PESAS ≥ 5, positive predictive value (PPV) was 31.7% (95% CI 27.6%–36.1%) and negative predictive value (NPV) was 97.6% (95% CI 96.1%–98.5%). The log‐likelihood ratio was significant (*p* < 0.001) and the Hosmer–Lemeshow statistic was insignificant (*p* = 0.342). Mortality rates for different PESAS values are shown in Figure 2 and compared to the original derivation cohort in Table 2. Observed mortality rates for lower‐acuity patients with PESAS ≤ 7 were uniformly lower in our validation cohort than in the derivation cohort. In both groups, however, PESAS = 10 was a clear inflection point at which risk for 30‐day mortality began to exceed 50%. Within the cohort, observed mortality rates were overall lower than those from the derivation cohort, and the observed‐to‐expected (O/E) ratio was 0.81 (95% CI 0.70–0.93). At the group level, a weighted least‐squares regression demonstrated a calibration slope of 1.04 (95% CI 0.90–1.17; *R* ^2^ = 0.955) and intercept of −4.25 (95% CI −7.65 to −0.86, Figure 3).

**FIGURE 2:**
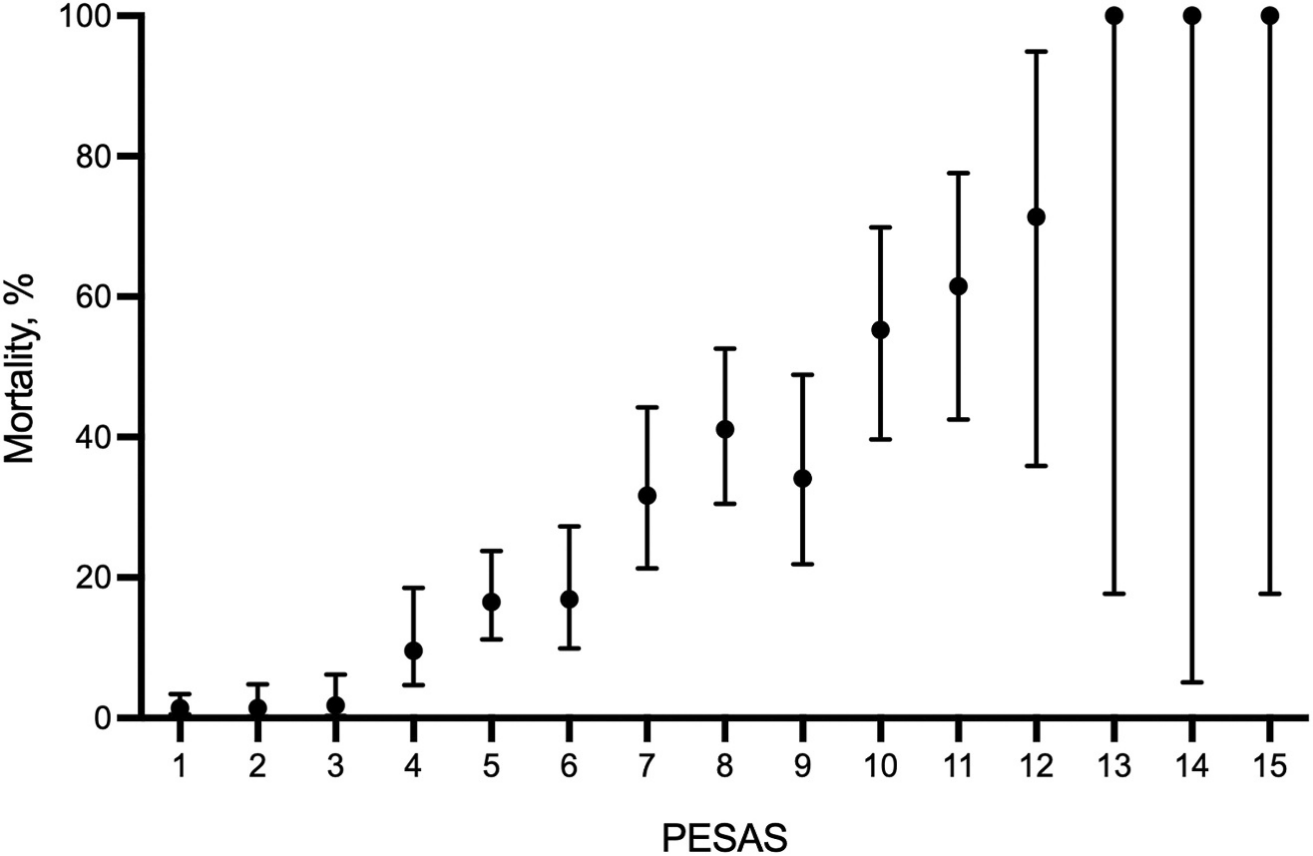
Observed mortality by PESAS. Mean values are presented with 95% Wilson confidence intervals. PESAS, Physiological Emergency Surgery Acuity Score.

**TABLE 2:** Observed 30‐day mortality by PESAS score.

PESAS	*n*	Mortalities	Observed mortality, % (95% CI)	Derivation cohort^a^ Mortality, %	O/E
1	296	4	1.4 (0.5–3.4)	3.1	0.45
2	147	2	1.4 (0.2–4.8)	5.7	0.25
3	113	2	1.8 (0.3–6.2)	9.1	0.20
4	73	7	9.6 (4.7–18.5)	14.4	0.67
5	133	22	16.5 (11.2–23.8)	20.4	0.81
6	71	12	16.9 (9.9–27.3)	25.6	0.66
7	60	19	31.7 (21.3–44.2)	34.2	0.93
8	73	30	41.1 (30.5–52.6)	40.6	1.01
9	44	15	34.1 (21.9–48.9)	45.9	0.74
10	38	21	55.3 (39.7–69.9)	55.3	1.00
11	26	16	61.5 (42.5–77.6)	58.3	1.05
12	7	5	71.4 (35.9–94.9)	64.5	1.11
13	2	2	100 (17.7–100)	51.6	1.93
14	1	1	100 (5.1–100)	100	1.00
15	2	2	100 (17.7–100)	100	1.00

*Note:* Sample size and mortality rates represent validation cohort unless otherwise specified. Confidence intervals represent 95% Wilson confidence intervals.

Abbreviations: CI, Confidence interval; O/E, Observed‐to‐expected ratio; PESAS, Physiological emergency surgery acuity score.

^a^ Derivation cohort: Sangji NF, Bohnen JD, Ramly EP, Velmahos GC, Chang DC, Kaafarani HMA. Derivation and Validation of a Novel Physiological Emergency Surgery Acuity Score (PESAS). World J Surg. 2017 Jul;41(7):1782–1789.

**FIGURE 3:**
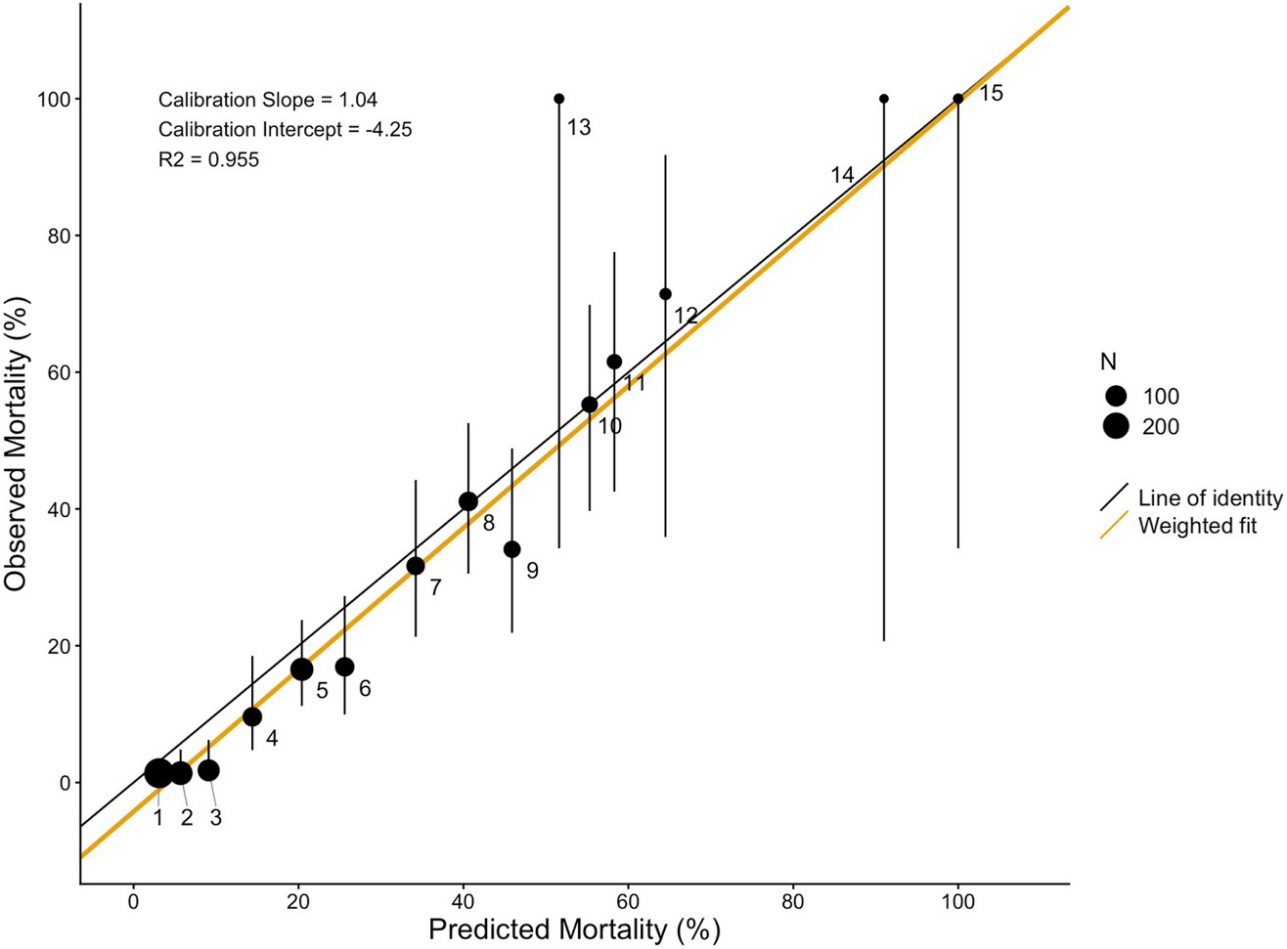
PESAS calibration. Calibration was performed at the level of the individual PESAS score for the outcome of 30‐day mortality using an ordinal least‐squares linear regression model of validation cohort observed risk against derivation cohort predicted risk, weighted for validation cohort sample size. Point size represents per‐score patient totals from the validation cohort, and points are presented with 95% Wilson confidence intervals. PESAS, Physiological Emergency Surgery Acuity Score.

PESAS discrimination for 30‐day mortality (AUROC 0.86) was superior to that of the ASA classification (AUROC 0.77, 95% CI 0.73–0.80), the Charlson Comorbidity Index (AUROC 0.59, 95% CI 0.54–0.64), and a combined ASA‐and‐CCI logistic model (AUROC 0.78, 95% CI 0.74–0.82; DeLong's *p* < 0.001 for all comparisons).

Decision curve analysis demonstrated that a PESAS‐guided strategy produced net benefit across threshold probabilities ranging from roughly 5%–50% (Figure 4). At threshold probabilities of 10%, 15%, and 25%, a PESAS‐guided strategy yielded net benefit of 0.101, 0.083, and 0.052, respectively. These net benefits values correspond to roughly 10, 8, and 5 additional high‐risk patients correctly identified per 100 (vs. treat‐none approach).

**FIGURE 4:**
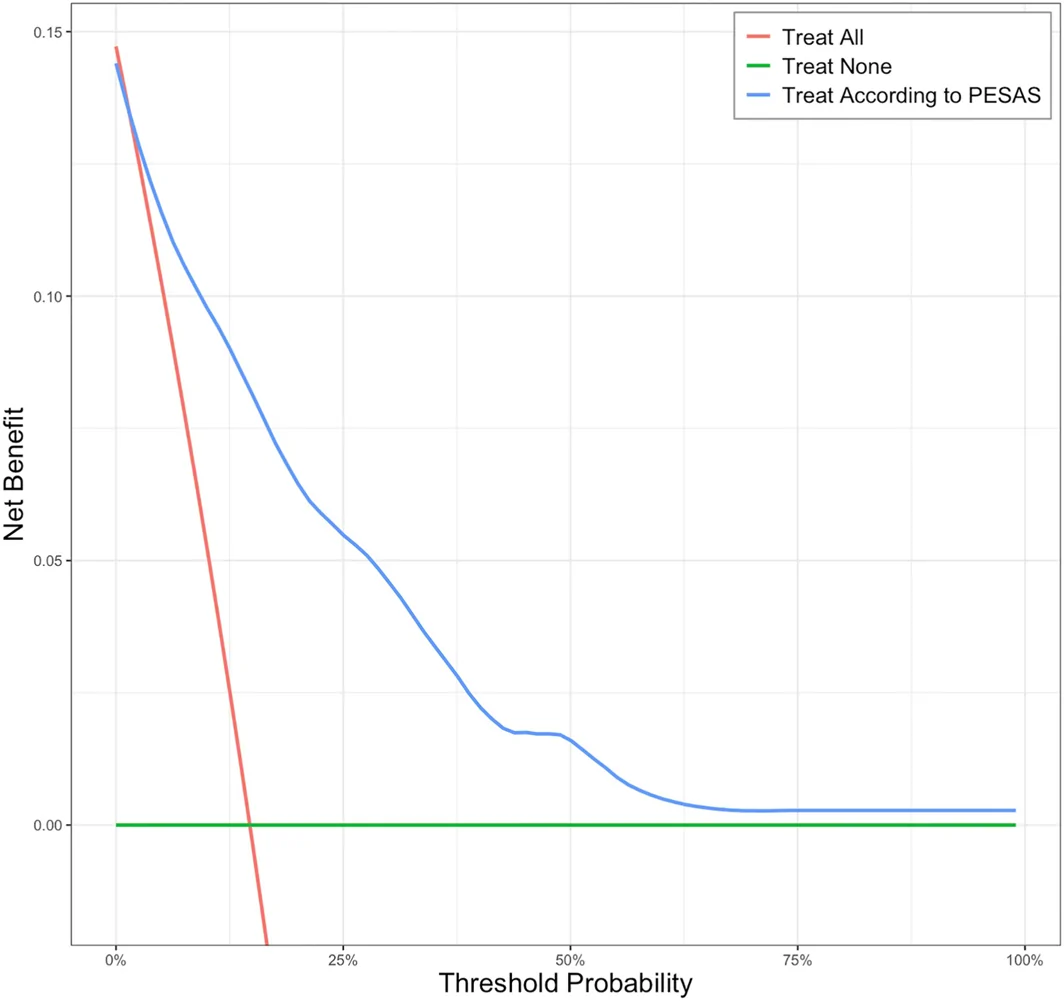
Decision curve analysis. A decision curve analysis is presented for the outcome of 30‐day mortality using a PESAS‐guided strategy across threshold probabilities from 0% to 100%. Net benefit is visualized for 3 strategies: treat all (red), treat none (green), and treat according to PESAS (blue). Prevalence of mortality within the validation cohort of *n* = 1086 was 14.7%. PESAS, Physiological Emergency Surgery Acuity Score.

To facilitate further interpretation, patients were grouped into three risk strata (PESAS 1–4, 5–9, and 10–15), and group mortalities were plotted alongside 95% confidence intervals (Figure 5A). Mortality increased in stepwise fashion across strata without overlap of confidence intervals, suggesting risk for 30‐day mortality was meaningfully separated at PESAS values of 5 and 10.

**FIGURE 5:**
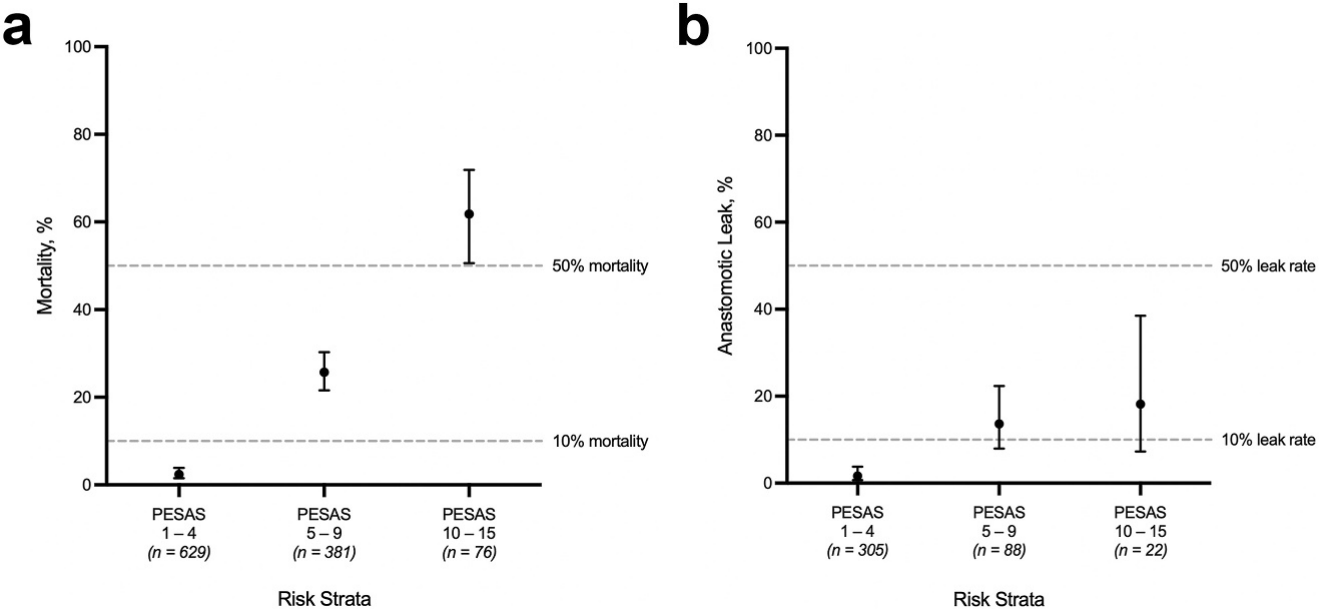
PESAS risk strata for study outcomes. Mean values are presented with 95% Wilson confidence intervals for outcomes of 30‐day mortality (A) and anastomotic leak (B). PESAS, Physiological Emergency Surgery Acuity Score.

Secondary analysis was conducted for the outcome of anastomotic leak. Among 414 patients who received a primary bowel anastomosis, 61 concurrently received an ostomy, including 29 with loop ileostomy (7.0%), 16 with end ileostomy (3.9%), and 16 with end colostomy (3.9%). There were 21 instances of anastomotic leak requiring reoperation (5.1%). Secondary analysis showed that PESAS had good discrimination for anastomotic leak with an AUROC of 0.82 (*p* < 0.001; 95% CI 0.73–0.90). The odds ratio per one‐point increase in PESAS was 1.43 (95% CI 1.24–1.67). The optimal predictive threshold for anastomotic leak was PESAS ≥ 7 with a sensitivity of 76.2% (95% CI 54.9%–89.4%), specificity of 82.0% (95% CI 77.9%–85.5%), PPV of 18.0% (95% CI 11.4%–27.2%), and NPV of 98.5% (95% CI 96.6%–99.4%). Model fit was acceptable with a significant log‐likelihood ratio (*p* < 0.001) and insignificant Hosmer–Lemeshow statistic (*p* = 0.859). In stratified subgroup visualization, patients with PESAS 5–9 had a higher leak rate compared to those with PESAS 1–4 (Figure 5B). There was no clear separation between the 5–9 and ≥ 10 strata, with considerable overlap of confidence intervals.

## 4 Discussion

Our findings represent the first large‐scale reporting of PESAS in patients specifically undergoing emergency laparotomy. We observed C‐statistics of 0.86 for the outcome of 30‐day mortality and of 0.82 for the secondary outcome of anastomotic leak. Optimal predictive thresholds occurred at PESAS ≥ 5 for 30‐day mortality and PESAS ≥ 7 for anastomotic leak. Score‐level calibration‐in‐the‐large for 30‐day mortality was reasonable, with O/E of 0.81, calibration slope of 1.04, and intercept of −4.50. Decision curve analysis demonstrated net benefit for 30‐day mortality across thresholds from roughly 5%–50%.

Studies applying widely adopted risk stratification scores to the emergency laparotomy population for the outcome of 30‐day mortality have yielded C‐statistics of 0.84–0.90 [8, 9, 10, 11, 12, 13, 14]. These studies have largely focused on the ACS‐NSQIP calculator, POTTER, and ESAS. Within our cohort, PESAS discrimination significantly exceeded that of the CCI and ASA classification combined, supporting performance of the score relative to common risk‐predictive comparators. Aside from mortality, fewer studies have evaluated the utility of scores for predicting anastomotic leak in emergency laparotomy. One prospective observational study found the Hajibandeh Index had a C‐statistic of 0.74 in predicting anastomotic leak after emergency laparotomy, which outperformed the ACS‐NSQIP calculator and others [15]. The Hajibandeh Index was designed to predict the presence of intraperitoneal contamination and, like PESAS, uses only objective variables derived from laboratory studies [16]. The good discriminatory performance of these scores represents how physiologic acuity influences anastomotic leak more than factors like demographics or comorbidity, which are common components of many risk prediction models. Our findings suggest PESAS offers comparable and potentially greater discriminatory power for predicting anastomotic leak relative to similar scores, although our leak analysis included only 21 events. We recommend that future studies investigating this endpoint should include PESAS in comparisons.

Our data also suggests that the PESAS can be used to compare acuity and adjust risk between different groups of patients receiving emergency laparotomy. When comparing our mortality rates to the original derivation study, there seemed to be a natural cutoff at PESAS = 5 corresponding with the transition from lower to higher surgical risk [4]. There was also an apparent inflection point in both cohorts at PESAS = 10, where mortality began to exceed 50%. Visualization of these risk strata supported the concept of three distinct risk tiers because 95% confidence intervals did not overlap between groups. Results of calibration and decision curve analysis indicated that the derivation cohort PESAS risk estimates remained largely applicable within our validation cohort, with mild overprediction at lower scores that would warrant recalibration prior to using PESAS for individual probability estimates. When anastomotic leak was similarly visualized, PESAS ≥ 5 appeared associated with significantly higher risk for leak compared to PESAS ≤ 4, but this study was underpowered for more granular analysis of this outcome. A tier‐based approach to risk stratification works intuitively with PESAS because it is an ordinal rather than a continuous score. When applied retrospectively, PESAS may therefore enable meaningful outcome comparisons across cohorts, institutions, or larger data sets on the basis of preoperative physiologic acuity.

Decisional risk thresholds for performing laparotomy may vary by stakeholders and be influenced by a very wide variety of factors. In real‐world surgical practice, risk thresholds represent probabilistic cutoffs at which clinical equipoise surrounds the decision to operate, which could be operationalized as the range across which the preoperative harm‐to‐benefit ratio of laparotomy is unclear [17, 18]. In the case of emergency laparotomy, this happens to be a very wide range of thresholds. Data exists to inform the upper and lower limits of risk between which clear equipoise exists. At the lower end of the risk spectrum, the National Emergency Laparotomy Audit (NELA) defines “high risk” as a predicted 30‐day mortality of ≥ 5%, with some believing this threshold ought to trigger escalation to operative care rather than continued nonoperative management [19]. At the higher end of the risk spectrum, futility is difficult to predict. Among patients with NELA‐predicted 30‐day mortality ≥ 50%, 90‐day survival ranged from 43% at 50%–59% predicted risk, to 17% at ≥ 80% predicted risk [20]. Around 35% of older adults with extreme preoperative risk (≥ 75% predicted 30‐day mortality) have been shown to die within 48 h of surgery, implicating that nonbeneficial surgery is common in the extreme‐risk subgroup and that nonoperative or palliative care pathways may align better with patient goals [21]. However, even in extremely low‐ or high‐risk patients, clear surgical indications typically still warrant operation, and the emergency laparotomy decision requires consideration of much more information than mortality risk alone, such as frailty or underlying pathology [21, 22]. Accordingly, guidelines frame the high‐risk laparotomy decision as individualized and driven by goals‐of‐care, with risk scores such as PESAS serving an informatory rather than confirmatory role in decisions [23, 24]. Our results support PESAS as a valid tool for informing goal‐concordant shared decision‐making in emergency surgery.

We found the optimal threshold for predicting anastomotic leak was slightly higher than that observed for 30‐day mortality. Additionally, upon data visualization, risk for leak among patients with PESAS ≥ 10 was not clearly elevated compared to patients in the PESAS 5–9 stratum. When bowel resection is performed, sicker patients are more likely to experience mortality before anastomosis, or to receive an ostomy rather than a primary anastomosis. The patients in our anastomotic subgroup with PESAS ≥ 10 were surgeon‐selected to receive primary anastomosis and therefore probably represent the healthiest patients within that tier of data.

One specific benefit of PESAS relative to other predictive methods is that the score input is obtained solely from routine laboratory studies, in addition to the binary of mechanical ventilation. It leverages data that is readily available to clinicians and does not require invasive testing or impose delay. This implies that the PESAS could be a good candidate score for EMR integration to facilitate automated risk assessment when used alongside clinical decision support software. Automation may be easier with PESAS compared to predictive models which require subjective and qualitative variables, some of which rely upon practitioner judgment or patient participation. For example, PESAS retains integrity in situations where patients are unable to communicate or when past medical history is totally unknown. Automated risk assessment would greatly relieve human workload in analysis of large datasets. This could have future utility for purposes of quality benchmarking and financial compensation. In the current American healthcare landscape, hospital compensation is influenced strongly by case mix index (CMI) and weighted diagnosis‐related groups (DRGs) which are determined post hoc after treatments have been given. However, patients who share similar comorbidities and diagnoses can still differ significantly with respect to acuity. Acuity is incompletely captured by current federal reimbursement methods. PESAS characterizes risk before any operative interventions have occurred, and when applied retrospectively, the score demonstrates how postoperative outcomes align with preoperative risks. Incorporation of PESAS may allow for more accurate estimation of preoperative acuity, potentially enabling a more equitable reimbursement system for inpatient facilities and physicians.

Future research can take many directions. It is a priority to develop surgical risk scores which maintain validity in the nonoperative population. Besides ASA and ACS‐NSQIP, no widespread risk predictive models generalize to EGS patients managed nonoperatively [3]. Regarding the use of PESAS for anastomotic leak, future research could explore how PESAS correlates with surgeon preference for ostomy or primary anastomosis. Further study could also explore how higher scores perform for the prediction of leak after adjusting for selection bias. The ability of PESAS to predict morbidities beyond leak remains relatively unknown. Finally, further work is needed to characterize the predictive value of emergency surgery risk calculators in patient subpopulations and for specific operations, as this could lead to more refined models.

Limitations of the current study include the single‐center design and the exclusive application of our findings to patients undergoing emergency laparotomy. It remains unknown whether PESAS has predictive value for EGS patients managed nonoperatively, or for those undergoing only laparoscopic emergency general surgery procedures. We had low sample sizes for some PESAS scores, especially 12 and above, relative to the rest of the cohort. The secondary anastomotic leak analysis was underpowered, and findings should be interpreted as exploratory. For the outcome of anastomotic leak, only patients who received bowel resection and anastomosis were included, technical factors were not considered, and the subgroup is subject to bias related to operative decision‐making. Because the original PESAS publication provided no specific probability equation, we did not attempt reconstructing one, as recovering a patient‐level regression from a simplified score may be highly inaccurate [25]. Instead, we assessed calibration at the level of individual PESAS strata rather than individual patients, so our analysis does not guarantee calibration at the per‐patient level.

## 5 Conclusions

PESAS shows good discrimination, modest calibration‐in‐the‐large, and overall net benefit for predicting 30‐day mortality in patients undergoing emergency laparotomy for nontraumatic indications, although recalibration may help facilitate expanded use. In exploratory secondary analysis, PESAS also showed good discrimination for anastomotic leak. PESAS requires no subjective variables and therefore may be a good candidate score for large‐scale benchmarking and risk adjustment in emergency general surgery populations.

## Author Contributions

**Wade Hopper:** conceptualization, investigation, data curation, formal analysis, writing – original draft, writing – review and editing, methodology, validation, visualization, software. **Anthony J. Duncan:** conceptualization, investigation, data curation, writing – review and editing. **Mentor Ahmeti:** conceptualization, writing – review and editing, supervision, project administration.

## Funding

The authors have nothing to report.

## Ethics Statement

This study received Institutional Review Board approval (STUDY00004011). This work is currently not being reviewed and has not been accepted or published by any other journals. This work was presented on April 15, 2025, at the 2026 Annual Meeting of the Southwestern Surgical Congress in Tucson, AZ.

## Consent

A waiver of patient consent was obtained from the Institutional Review Board (STUDY00004011).

## Conflicts of Interest

The authors declare no conflicts of interest.

## Supporting information


Supporting Information S1

## Data Availability

The data that support the findings of this study are available from the corresponding author upon reasonable request.
